# Parental Perspectives on Sports Participation in Boys with Autism Spectrum Disorder Level 1 Following a Multicomponent Physical Activity Program: A Qualitative Study

**DOI:** 10.3390/children13091206

**Published:** 2026-09-07

**Authors:** Marina Conesa-Molina, María Dolores Grau, Ana Pablos, Laura Elvira

**Affiliations:** 1 Doctoral School, Catholic University of Valencia, San Vicente Mártir, 46001 Valencia, Spain; 2 Faculty of Psychology, Catholic University of Valencia, San Vicente Mártir, 46001 Valencia, Spain; lola.grau@ucv.es; 3 Faculty of Physical Activity and Sport Sciences, Catholic University of Valencia, San Vicente Mártir, 46900 Torrent, Spain; ana.pablos@ucv.es

**Keywords:** autism spectrum disorder, physical activity, barriers, parental perspectives, qualitative research, sports participation

## Abstract

**Highlights:**

**What are the main findings?**

- Parents commonly perceived their sons’ previous avoidance of physical activity as related to negative social and sporting experiences (e.g., bullying, exclusion, and experiences of failure) rather than to a generalized lack of interest in physical activity.
- Seven interconnected themes captured barriers (parental fears, social exclusion, methodological mismatches, untrained professionals, program scarcity, accessibility) and parent-perceived facilitators and outcomes (specialized instruction, small-group format, trained professionals, transfer to natural contexts) that, in parents’ accounts, supported their sons’ engagement in physical activity.

**What are the implications of the main findings?**

- Parents perceived that specialized sports programs delivered by professionals trained in autism, using small-group formats, visual supports and predictable structures, helped re-engage boys with ASD Level 1 who had previously withdrawn from physical activity.
- Policy and practice should address the “invisible population” gap by funding decentralized, family-centered programs that bridge mainstream and specialized services, while building capacity within mainstream sports organizations.

**Abstract:**

**Background**: Children with Autism Spectrum Disorder (ASD) Level 1 face barriers to sports participation that remain insufficiently understood. **Methods**: This qualitative study explored parental perspectives on barriers and facilitators to physical activity following participation in a multicomponent physical activity program (MCPAP). Semi-structured interviews were conducted with 17 parents (11 mothers, 6 fathers) of boys aged 8–12 years with ASD Level 1 who completed a 12-week MCPAP in Valencia, Spain. Data were analyzed using reflexive thematic analysis. **Results**: Seven themes were identified, capturing both barriers and facilitators: (1) parental fears and previous negative experiences; (2) parents’ accounts of their sons’ avoidance of physical activity in relation to bullying and social exclusion; (3) methodological barriers linked to non-adapted teaching approaches; (4) challenges with non-specialized professionals; (5) limited availability of programs specifically designed for children with ASD Level 1; and (6) location and accessibility concerns; and (7) parent-perceived facilitators and outcomes associated with the MCPAP, including transfer to natural contexts. **Conclusions**: Parents described children with ASD Level 1 as an “invisible population” with regard to existing sports opportunities. Parents further perceived that specialized programs with trained professionals, adapted methodologies, and small-group formats supported their sons’ sustained engagement in physical activity. These findings reflect parental interpretations and were not triangulated with standardized outcome measures.

## 1. Introduction

Physical activity participation offers numerous benefits for children with Autism Spectrum Disorder (ASD), including improvements in motor skills, social functioning, behavioral regulation, and overall quality of life [1,2,3,4]. Despite these documented benefits, children with ASD participate in physical activity at significantly lower rates than their typically developing peers [5,6,7]. This disparity is particularly concerning given that physical inactivity contributes to elevated rates of obesity and associated health complications in this population [8,9].

The barriers to physical activity participation in children with ASD are multifaceted and occur at individual, social, and environmental levels [10,11]. At the individual level, motor coordination difficulties, sensory sensitivities, and challenges in social communication can impede engagement in traditional sports settings [12,13]. Social barriers include negative peer interactions, bullying, and lack of understanding from coaches and instructors [14,15,16]. Environmental barriers encompass limited availability of adapted programs, inadequate professional training, and accessibility issues [17,18].

Children with ASD Level 1, as described in the broader diagnostic profile, are characterized by requiring support while typically demonstrating relatively preserved cognitive and language abilities [19], occupying a unique position within the autism spectrum.

Nevertheless, impairments in social communication are a core feature of ASD and include difficulties with pragmatics and the contextual use of language. Evidence points to deficits in conversational exchange, including difficulties with topic maintenance, perseveration, and providing relevant contributions during dialogue [20], which can affect the quality of peer interaction even when structural language abilities are intact. Comprehension of non-literal language, such as metaphor and idiom, may also be affected in some children with ASD, particularly when utterances are highly context-dependent or when co-occurring language difficulties are present [21]. These limitations affect core areas of development, such as socio-emotional adjustment and the quality of interpersonal relationships.

These children often participate in mainstream educational settings and community activities, yet their support needs may be overlooked or misunderstood [22,23]. The lack of visible impairment can lead to unrealistic expectations and inadequate accommodation, creating what some researchers have termed an “invisible disability” [23], particularly when intellectual and language abilities appear preserved. To date, limited research has specifically examined the physical activity experiences of children with ASD Level 1, with most studies aggregating participants across the spectrum or focusing on those with more significant support needs [10,11,17,24].

Understanding parental perspectives is crucial for identifying barriers and developing effective interventions, as parents serve as gatekeepers to their children’s activity opportunities and possess intimate knowledge of their children’s needs and preferences [24,25]. Qualitative research provides an essential methodology for capturing the nuanced experiences of families navigating the complex landscape of sports participation for children with ASD. Therefore, this study aimed to explore parental perspectives on barriers and facilitators to sports participation in boys with ASD Level 1 following their involvement in a multicomponent physical activity program specifically designed for this population.

## 2. Materials and Methods

### 2.1. Research Design

This study employed a qualitative descriptive design using semi-structured interviews to explore parental perspectives on sports participation barriers and facilitators for boys with ASD Level 1. The research was underpinned by an interpretivist paradigm, recognizing that participants construct meaning through their lived experiences [26]. Reflexive thematic analysis, as outlined by Braun & Clarke [27,28], was selected as the analytical approach due to its flexibility and suitability for identifying patterns of meaning across qualitative datasets. For consistency, the term “physical activity” is used throughout the manuscript to refer to any bodily movement that produces energy expenditure, while “sports participation” and “sport” are used interchangeably to refer to structured, rule-based physical activities practiced in organized or semi-organized settings.

Ethical approval was obtained from the institutional ethics committee prior to data collection. The study adhered to the Consolidated Criteria for Reporting Qualitative Research (COREQ) guidelines [29].

### 2.2. Participants

Participants were parents of boys who had completed the MCPAP. All 22 families whose sons had completed the program were invited to participate, and the first author contacted the primary caregiver of each family to introduce the study. Purposive sampling was applied to the composition of the resulting sample, with the aim of ensuring representation of both mothers and fathers with varied experiences (e.g., children’s age within the 8–12 range, coexistence of parents in the household, and previous sports participation). When more than one caregiver was eligible, the family decided which parent would take part. Of the 22 invited families, 18 agreed to participate and were interviewed; the remaining four families declined. One of the 18 audio recordings was of insufficient quality to be transcribed and was therefore excluded from the analysis, yielding a final analytic sample of 17 parents. Inclusion criteria required participants to (a) be a biological, step, or adoptive parent of a child with ASD Level 1 who had completed the MCPAP; (b) spend regular time with the child; and (c) be capable of providing informed consent.

Boys of participating parents met the following criteria: (a) aged 8–12 years; (b) a clinical diagnosis of ASD Level 1 established by a licensed clinician according to DSM-5 criteria [19] prior to enrollment, supported by the Autism Diagnostic Interview-Revised (ADI-R) and the Autism Diagnostic Observation Schedule (ADOS), with the Level 1 designation (i.e., “requiring support”) confirmed through these assessments; and (c) completion of the 12-week program.

For all participating children, ADI-R and ADOS information was obtained retrospectively from existing clinical reports held by the families; no additional diagnostic procedures were performed for the purpose of this study. Cognitive and language abilities were not directly assessed within the present study, and the description of children with ASD Level 1 as presenting relatively preserved cognitive and language abilities therefore refers to the general diagnostic profile rather than to measures obtained in this sample. Children were excluded if they had: (a) experienced bone fractures within the previous six months, (b) a clinical diagnosis of anxiety disorder requiring anxiolytic medication, or (c) current involvement in organized competitive sports (e.g., federated team sports, scheduled league competitions) at the time of recruitment, to minimize the influence of pre-existing training routines on the program’s effects.

**Figure 1:**
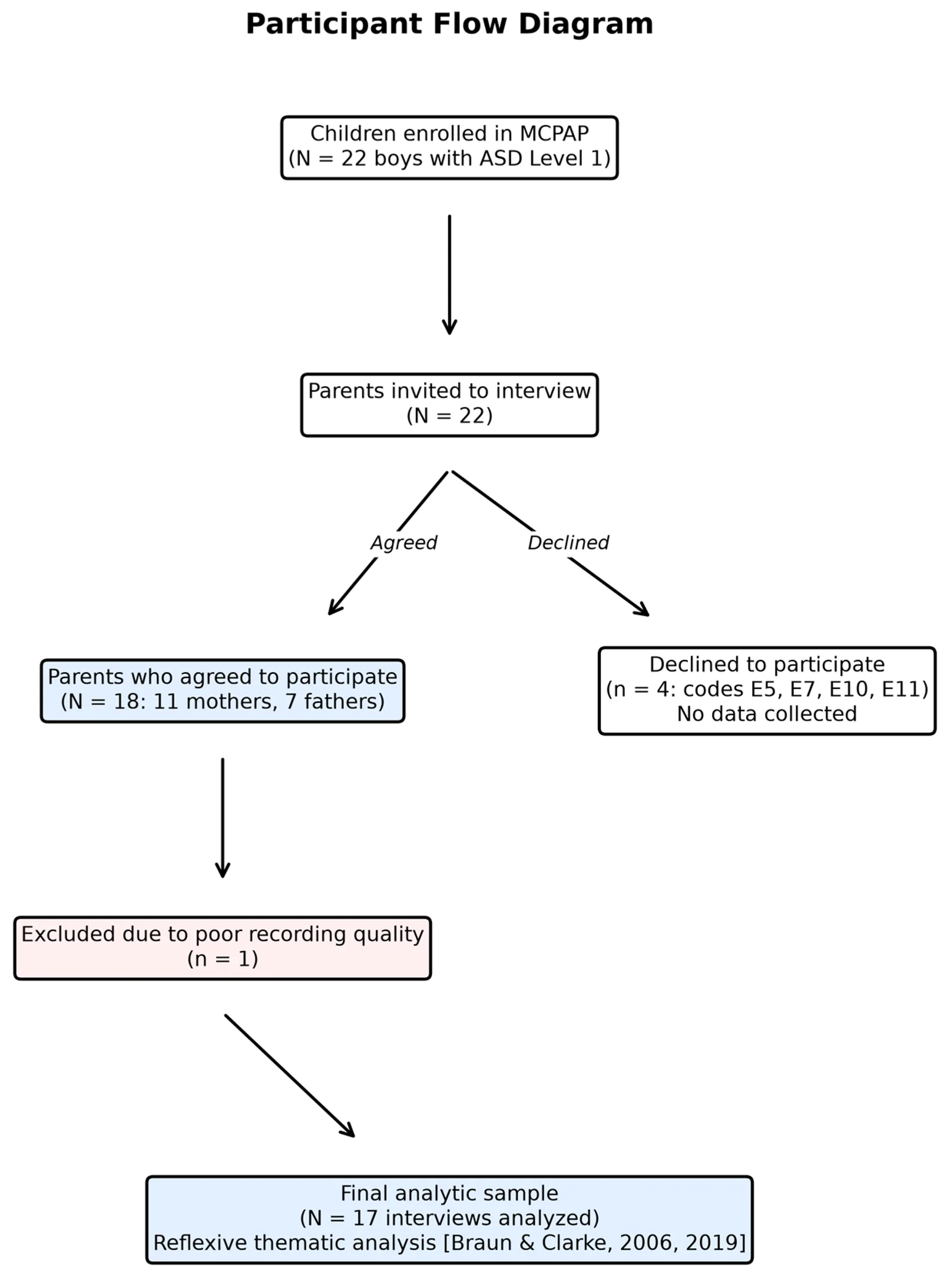
Participant flow diagram. The thematic analysis stage was based on Braun & Clarke [27,28].

The final analytic sample comprised 17 parents: 11 mothers (M_age_ = 45.45, SD = 3.36) and 6 fathers (M_age_ = 47.50, SD = 2.26). The participant flow from enrollment in the MCPAP through inclusion in the present analysis is shown in Figure 1. Detailed demographics and child characteristics are presented in Table 1. Because eligibility required completion of the 12-week program, the sample may over-represent families with more positive program experiences, an issue we return to in the Limitations section.

### 2.3. Context: The Multicomponent Physical Activity Program

The multicomponent physical activity program (MCPAP) was implemented across three consecutive editions between October 2023 and May 2025 in Valencia, Spain (first edition: 2 October–20 December 2023; second edition: 21 March–6 June 2024; third edition: 2 March–19 May 2025). Parents of children who completed the MCPAP in any of the three editions were interviewed in June 2025, approximately 1, 12, and 18 months after the completion of the third, second, and first editions, respectively. The program was designed specifically for children with ASD Level 1 and incorporated evidence-based strategies for autism-inclusive physical activity instruction [14,30,31]. Key program components included: (a) small-group format with up to 8 children per group; (b) two trained professionals per group, resulting in a professional-to-participant ratio ranging from 2:6 to 2:8 (mean ratio across editions: ~2:7, including typically developing peers); (c) the inclusion of 2 neurotypically developing peers per group (6 peers across the three editions) to support social interaction and peer modeling; (d) structured, predictable session formats based on the TEACCH framework [32]; (e) visual supports and adapted communication strategies; (f) activities targeting aerobic capacity, strength, motor skills, balance, and executive functions through motor-cognitive dual-tasks; and (g) progressive muscle relaxation integrated as a narrative-based cool-down to foster emotional regulation.

The decision to maintain small group sizes and a 2:6 to 2:8 professional-to-participant ratio was an adaptation of the TEACCH principle of individualization, intended to sustain attention, facilitate social interaction, and minimize distractions and anxiety-provoking elements that can arise in larger groups. The presence of two professionals per session allowed individualized behavioral support when required without interrupting ongoing activities, thereby maintaining program continuity and group regulation.

All intervention sessions followed a standardized sequence: introduction and visual schedule review (~5 min), warm-up (~10 min), physical and motor module (20–30 min), cognitive training module (20–30 min), cool-down (~10 min), and closure with token economy review (~5 min). Sessions lasted 60 min and occurred twice weekly for 12 weeks. To be included in the analysis, participants were required to attend at least 75% of scheduled sessions; attendance was monitored weekly.

### 2.4. Data Collection

Semi-structured interviews were conducted in June 2025, approximately one month after the end of the third edition of the MCPAP. This interval allowed parents sufficient time to reflect on the program experience and to observe any post-program changes in their children’s physical activity behaviors. The original language of the interviews was Spanish (Spain). All quotations presented in this manuscript were translated into English for publication by a bilingual research team and independently checked by a second bilingual person against the original Spanish transcripts and audio recordings, with the aim of preserving meaning rather than literal wording. An interview guide was developed based on the research aims and relevant literature, covering six domains: (1) general family and child information; (2) experiences within the MCPAP; (3) observed changes in the child; (4) impact on family dynamics; (5) program evaluation; and (6) final comments and suggestions. The guide was pilot-tested with two parents not included in the final sample and refined for clarity accordingly. The questions were open-ended to allow parents to freely express their experience during the program; the full interview guide is publicly available at https://osf.io/zdvhb/ (accessed on 8 August 2026) (see Data Availability Statement).

Interviews were conducted via the Microsoft Teams online videoconferencing platform by an external researcher trained in qualitative methods, who had no prior relationship with the program or the participants. Interviews lasted between 45 and 90 min. All interviews were audio-recorded with participant consent and transcribed verbatim. Field notes were taken during and immediately following each interview to capture contextual observations and initial analytical reflections.

### 2.5. Data Analysis

Data were analyzed using Braun & Clarke [27,28] six-phase reflexive thematic analysis. Phase one involved repeated reading of transcripts to achieve data familiarization. In phase two, initial codes were generated systematically across the dataset. Phase three involved collating codes into potential themes, with attention to both semantic and latent content. During phase four, themes were reviewed against coded extracts and the full dataset to ensure coherence and distinctiveness. Phase five involved defining and naming themes to capture their essence. Finally, phase six comprised producing the analytical narrative presented in the results section.

Initial coding and data familiarization were conducted by the first author. A second researcher independently coded a subset of transcripts (approximately 25%) to develop and challenge the first author’s interpretations, in line with the reflexive orientation of the analysis; rather than as a procedure for resolving disagreement between coders, this collaborative coding was used to challenge assumptions, bring alternative readings to light, and refine emerging themes. The first author’s prior involvement in the program was consistently viewed as a contextual resource to be examined reflectively, rather than as a primary analytical lens. The subsequent development of themes, their naming, and their analytical refinement were carried out collaboratively by the research team, and the first author’s interpretations were explicitly discussed and challenged during research meetings. An audit trail was created to document the analytical decisions made throughout the coding and theme development process.

### 2.6. Trustworthiness

Several strategies were employed to enhance the trustworthiness of the findings [33,34]. To strengthen credibility, member checking was conducted with two parents (one mother and one father, purposively selected among those available at the close of the analysis, given the study’s sample size); both confirmed that the themes resonated with their experiences, and minor refinements to theme wording were incorporated based on their feedback. Peer debriefing sessions were held with two members of the research team who had not been involved in the program’s implementation; these sessions examined emerging codes, challenged interpretations, and refined the thematic structure.

Dependability was supported by the maintenance of an audit trail documenting the analytical decisions made throughout the process, including the code book, the code-to-theme matrix, and the chronology of theme refinement [35].

The first author is the program facilitator, which constitutes an insider position with respect to the research context, and was also involved in the initial coding and in some interview accounts that explicitly evaluate her own approach. To address this, the author maintained a reflexive journal throughout the analysis, documenting preconceptions, emotional reactions, and potential biases related to prior involvement with the families. The wider research team, and particularly the third author who independently coded a subset of transcripts, explicitly challenged interpretations that could be perceived as fostering the program. All interviews were conducted by an external researcher with no prior relationship with the program or the families, separating data collection from program delivery.

## 3. Results

Analysis revealed seven interconnected themes describing barriers and facilitators to sports participation in children with ASD Level 1: (1) Subjective Barriers Rooted in Parental Fears, (2) Children’s Rejection as Response to Social Exclusion, (3) Methodological Barriers in Conventional Settings, (4) Challenges with Non-Specialized Professionals, (5) Scarcity of Specific Programs for ASD Level 1, and (6) Location and Accessibility Concerns, and (7) Facilitators and Positive Outcomes Associated with the MCPAP. Themes and subthemes are summarized in Table 2. Participant identifiers (E1–E22) are used to maintain confidentiality while indicating the breadth of perspectives represented.

### 3.1. Subjective Barriers Rooted in Parental Fears

Parents described significant apprehension about enrolling their children in sports activities, shaped by previous negative experiences and concerns about potential adverse outcomes. These fears constituted substantial psychological barriers that often preceded objective assessment of program suitability.

**Fear of Failure and Rejection**. Many parents harbored concerns that their children would be unable to meet the demands of sports activities, leading to frustration and rejection. One mother explained:

“My son struggles a lot with sports… my fear was he’d start and then say, ‘mom, I don’t want to go anymore.”

(E20)

This anticipatory anxiety was often rooted in previous attempts at sports participation that had ended negatively. Parents described a pattern of initial enthusiasm giving way to withdrawal:

“I worried he would become disenchanted; he didn’t want to do any exercise.”

(E8)

For some families, this apprehension extended to the very first session, with parents describing their emotional state at the door:

“I went with a bit of fear, to see how he would behave…”

(E13)

**Fear of Social Difficulties**. Parents expressed particular concern about their children’s social vulnerability in sports contexts. The combination of social communication differences and the interactive nature of team sports created significant parental anxiety:

“A bit scared about integrating; he has significant overweight… doesn’t feel integrated because they’re not at his motor level.”

(E18)

Parents anticipated bullying and mockery, recognizing their children’s heightened sensitivity to peer rejection. These anticipated social risks, in some cases, were sufficient to deter enrollment altogether:

“Never managed to introduce him to any group sport; he didn’t want monitors.”

(E13)

**Fear of Incomprehension**. Parents worried that coaches and other adults would not understand their children’s needs or behavioral presentations. This concern shaped program selection from the outset:

“We looked at a gymnastics facility… and we could not find an activity that precisely met his needs.”

(E4)

This concern extended to the instructional environment, where parents anticipated that their children would be unable to follow directions and would be perceived negatively. In several cases, this perception was confirmed rather than anticipated, as described in the following theme.

### 3.2. Children’s Rejection as Response to Social Exclusion

A central finding of this study was the distinction parents drew between their sons’ apparent disinterest in physical activity and what they perceived as aversion resulting from negative social experiences. Parents consistently interpreted their children’s avoidance of sports as related to experiences of exclusion, bullying, and failure, although this does not exclude possible interactions with autism-related characteristics, motor difficulties, sensory factors, or communication demands.

**Bullying and Peer Rejection**. Parents identified bullying during sports activities as a primary contributor to their children’s subsequent avoidance. The competitive and social nature of sports environments appeared to amplify opportunities for peer rejection:

“He complained they laughed at him, had to quit after 2 months.”

(E4)

“He doesn’t have fun with sports… soccer, basketball, nothing… none of that works.”

(E16)

“We tried boxing… he signed up on his own, and since he couldn’t go with his brothers either, it was harder for him to adapt and he ended up quitting.”

(E17)

According to parent reports, these experiences of mockery, whether related to motor skill differences, social behavior, or physical characteristics, contributed to lasting negative associations with sports participation.

**Accumulated Negative Experiences.** Multiple failed attempts at sports participation created cumulative frustration that intensified children’s resistance. One father summarized a typical trajectory:

“We tried rugby, but the instructions weren’t understood, teamwork mechanics weren’t grasped.”

(E3)

From the parents’ perspective, these repeated experiences led children to generalize their negative expectations across all sports contexts, making subsequent attempts at engagement increasingly difficult.

**Rejection of Context, Not Activity**. Importantly, parents observed that their children’s apparent rejection of physical activity was actually a rejection of the social circumstances surrounding it. Following participation in the MCPAP, many children demonstrated enthusiasm for physical activity and sought continued involvement in sports:

“Now he’s doing karate… same profile as Z and doing well.”

(E4)

“He’s enthusiastic and continued; now in athletics classes.”

(E8)

“Now he’s into soccer.”

(E18)

These observations supported the interpretation that children’s previous rejection reflected avoidance of negative social experiences rather than inherent disinterest in physical activity.

### 3.3. Methodological Barriers in Conventional Settings

Parents described how conventional sports instruction methods created significant barriers for their boys with ASD Level 1. The mismatch between standard teaching approaches and children’s learning needs emerged as a consistent theme.

**Adapted Communication and Structured Delivery.** The verbal complexity and implicit expectations characteristic of conventional sports instruction posed challenges for children with ASD Level 1, as illustrated by the failed rugby attempt described earlier (E3).

Parents emphasized the need for simple, clear, and direct instructions that their children could readily understand and follow. One parent contrasted his son’s rejection of other sports with his engagement in a different format:

“He doesn’t do any sport. But this yes, very happy. It was like games… directed games that schools don’t do.”

(E16)

The lack of structure and predictability in conventional programs compounded these communicative challenges, creating anxiety and confusion for children accustomed to routine. Several parents also noted that heterogeneity of skill level within a group could be problematic:

“I would do an initial assessment and make sure children in the group have a similar level, so that some children are not waiting for others; with ASD children, waiting makes them desperate, and the one who can’t keep up gets nervous and wants to leave.”

(E22)

Indoor facilities were also preferred by some families, particularly for the winter months:

“It was difficult to do the activity because it was an open space, and the location where the program was held was not made clear… maybe for the winter, an indoor sports hall would have been better.”

(E14)

**Group Size and Attention**. Large group formats typical of community sports programs limited individualized attention and created overwhelming social environments. One parent summarized a preference shared by many:

“Smaller groups meant more attention… larger groups are more complicated for them to follow instructions.”

(E3)

Parents valued the small group format of the MCPAP, which allowed for appropriate monitoring and support while maintaining opportunities for peer interaction.

### 3.4. Challenges with Non-Specialized Professionals

Parents consistently highlighted the critical importance of professionals trained to understand and support children with ASD Level 1. Experiences with untrained professionals had contributed to previous failures, while the specialized expertise of MCPAP instructors was identified as a key success factor.

**Understanding Behavioral Presentations**. Parents valued professionals who recognized that challenging behaviors often reflected underlying difficulties rather than willful defiance:

“My son handles frustration very badly… X had exceptional management skills, let him calm down then reintegrated him.”

(E15)

This understanding enabled appropriate responses that supported children’s emotional regulation rather than escalating difficulties.

**Adapted Communication and Redirection**. Parents praised the MCPAP instructor’s ability to communicate effectively and redirect children when needed:

“X understood them and explained everything well, if something didn’t work, stopped and re-explained with much calm.”

(E16)

“Small groups and people who know how to treat them… X’s work was very important in redirecting.”

(E22)

**Expertise as Essential**. Parents consistently identified professional expertise in ASD as a critical factor distinguishing success from unsuccessful sports experiences:

“Key that X has excellent understanding of the autism spectrum… huge difference when someone knows how to manage their behavioral peaks.”

(E21)

This expertise encompassed both theoretical knowledge of ASD and practical skills in implementing supportive strategies, including proactive redirection rather than allowing children to disengage:

“Normally, in most programs, when children do something and want to leave, the instructor lets them go and doesn’t insist. X’s work was very important in redirecting them, bringing them back to the program, making them feel included.”

(E22)

Not all participants, however, experienced the program as uniformly positive. One mother reported that her son, who had a general dislike of sports, found attendance burdensome and that an indoor facility would have been preferable during the winter months (E14). This observation underscores the need for an individualized assessment of children’s relationship with physical activity prior to enrollment and for flexible implementation contexts that can accommodate varying levels of initial engagement.

### 3.5. Scarcity of Specific Programs for ASD Level 1

Parents repeatedly emphasized the lack of appropriate sports programs for children with ASD Level 1, describing their children as falling between service categories designed for either typically developing children or those with more significant disabilities.

**The “Invisible Population”.** Parents described their children as occupying an ambiguous space, too affected for mainstream programs yet too capable for programs designed for individuals with more significant needs:

“Very beneficial these initiatives for ASD level 1 children… a very invisible population. All programs are focused on severe disability, they don’t fit normative or severe populations, leaving them in a kind of limbo.”

(E22)

This invisibility, as described by parents, resulted in a service gap that left families without appropriate options.

**Lack of Systemic Guidance.** Parents expressed feelings of being unsupported in their search for suitable activities:

“Very abandoned when taking him to sports, we set aside the medical part… it is very complicated for a family to find a comfortable environment.”

(E1)

“There are options, but everything is very limited…”

(E13)

The burden of finding appropriate programs fell entirely on families, who often lacked guidance or resources.

**Need for Continued Initiatives.** Parents expressed a strong desire for the continuation and expansion of programs like the MCPAP:

“Next year, some activity… a group designed for ASD Level 1…”

(E9)

“I would love for the program to return. It was very positive for us and he really liked it; these kinds of programs should be offered more often in schools, during leisure time, even on the weekend, you know, to create these leisure spaces.”

(E12)

“It was a bit short… we need more time or that there was continuity.”

(E3)

### 3.6. Location and Accessibility Concerns

Beyond the limited availability of programs specifically designed for children with ASD Level 1, parents emphasized that even when such programs existed, geographic and logistical factors often determined whether families could realistically access them. Transportation challenges, time constraints associated with multiple therapy appointments, and the children’s difficulties with transitions made proximity to home or school a decisive factor in program uptake.

Parents described how the absence of programs in their immediate neighborhoods effectively negated the benefits of any specialized program that did exist:

“If it could be offered in several places at once… for example, one group working at the San Jose School, another at the Catholic University of Torrente gym… for convenience…”

(E4)

“The only part that was complicated was that the place was a bit far from us…”

(E17)

Several parents (E4, E9, E12, E13) reported being left with the choice between long commutes or no participation at all. Parents expressed hope that similar programs could be decentralized and offered in more locations, including schools and community sports clubs, to facilitate equitable access.

### 3.7. Facilitators and Positive Outcomes Associated with the MCPAP

Beyond the barriers identified in conventional settings, parents described the elements of the MCPAP that facilitated engagement and the outcomes they perceived in their children during and after the program. Four subthemes captured these parent-reported facilitators and outcomes: (a) behavioral and emotional regulation, (b) transfer to school and natural contexts, (c) reduced avoidance and continued engagement, and (d) broader well-being and family impact.

**Behavioral and Emotional Regulation**. Several parents described what they perceived as noticeable improvements in their children’s self-regulation and overall emotional state during the program period. One mother summarized a sustained change in her son’s affect:

“We noticed he was better. We noticed he was calmer.”

(E1)

The relaxation component at the end of each session was particularly valued:

“The last part they did was relaxation; they also paid attention, were focused, and the truth is that those last five or ten minutes were very good for them.”

(E17)

Several parents described concrete reductions in what they identified as challenging behaviors:

“At the beginning he had slightly more aggressive behaviors, and on the days he went to the program he was more relaxed. Because he had discharged energy, the episodes were reduced.”

(E17)

One parent reported a striking episode of emotional regulation in which her son, having become dysregulated at school, was taken to the MCPAP activity as an alternative to another appointment; in her account, the child finished the session regulated and calm:

“It was therapeutic, he left better than from the therapies themselves… he came out regulated, calm, happy, talking about what happened there; he left so much more regulated, so much more.”

(E21)

Transfer to School and Natural Contexts. Parents also reported changes in their children’s behavior in school settings, suggesting from their perspective that gains from the program extended beyond the activity itself. One parent described improvements reported directly by the school teacher:

“Regarding his hands, his manual abilities have improved. At school, during an activity he was more attentive and concentrated when receiving the ball, and the teacher told us they had noticed a change in that regard and that he was more skillful.”

(E2)

Another parent described how she observed the transfer of gains to the school playground:

“He went from not playing at all in the schoolyard to playing tag, to running and playing with his classmates in the schoolyard, as a result of the experience he had…”

(E20)

A particularly important form of transfer involved parental mediation of program strategies into the school context. One mother explained how she shared the MCPAP approach with her child’s physical education teacher, which she felt led to improvements in the school setting:

“I tried to explain a bit to the teacher how X did it, and the truth is that it went very well. At school we have seen that it has improved.”

(E13)

Reduced Avoidance and Continued Engagement. A consistent outcome reported across families was a reduction in fear and avoidance that had previously characterized their children’s relationship with physical activity. One mother noted:

“He has always been very sedentary; since then, he has taken up sport a bit more, with more motivation. Now, for example, he’s into soccer… Yes, he has lost a bit of fear.”

(E18)

Several children expressed explicit desire to continue:

“Yes, he went very happy; more than once he asked, ‘aren’t we going back to sports with X?’ Because he liked it…”

(E15)

“He is super motivated; he has attended competitions; he has won some of them… they don’t put him in special needs groups or anything like that; he goes with the rest of the group and adapts very well.”

(E19)

Broader Well-being and Family Impact. Finally, parents described broader changes in their children’s well-being and family functioning. Parent-reported improvements in sleep were reported:

“He slept much better.”

(E18)

Improvements in communication and language use were also noted:

“It generated more spontaneous language—in his case, most of it is echolalia—but it generated more spontaneous language, also affective language, because they built a bond among themselves.”

(E21)

“He is starting now to express things that he did not express before.”

(E22)

Several parents described the unexpected formation of a peer-support network among mothers:

“It was like a therapy group for mothers of children with ASD Level 1. Because we talked, we vented, we talked freely.”

(E20)

Together, these accounts suggest that the combination of adapted instruction, structured delivery, small group format, and professional expertise was perceived by parents as supporting their sons’ regulation, social engagement, and motivation, and as contributing to perceived changes that extended into natural contexts. It is important to note that these outcomes reflect parental perception rather than standardized measurement; nonetheless, parents are uniquely positioned to detect subtle changes in affect, motivation, and behavior across settings.

### 3.8. Conceptual Model of the Trajectory from Negative to Positive Sports Experiences

**Figure 2:**
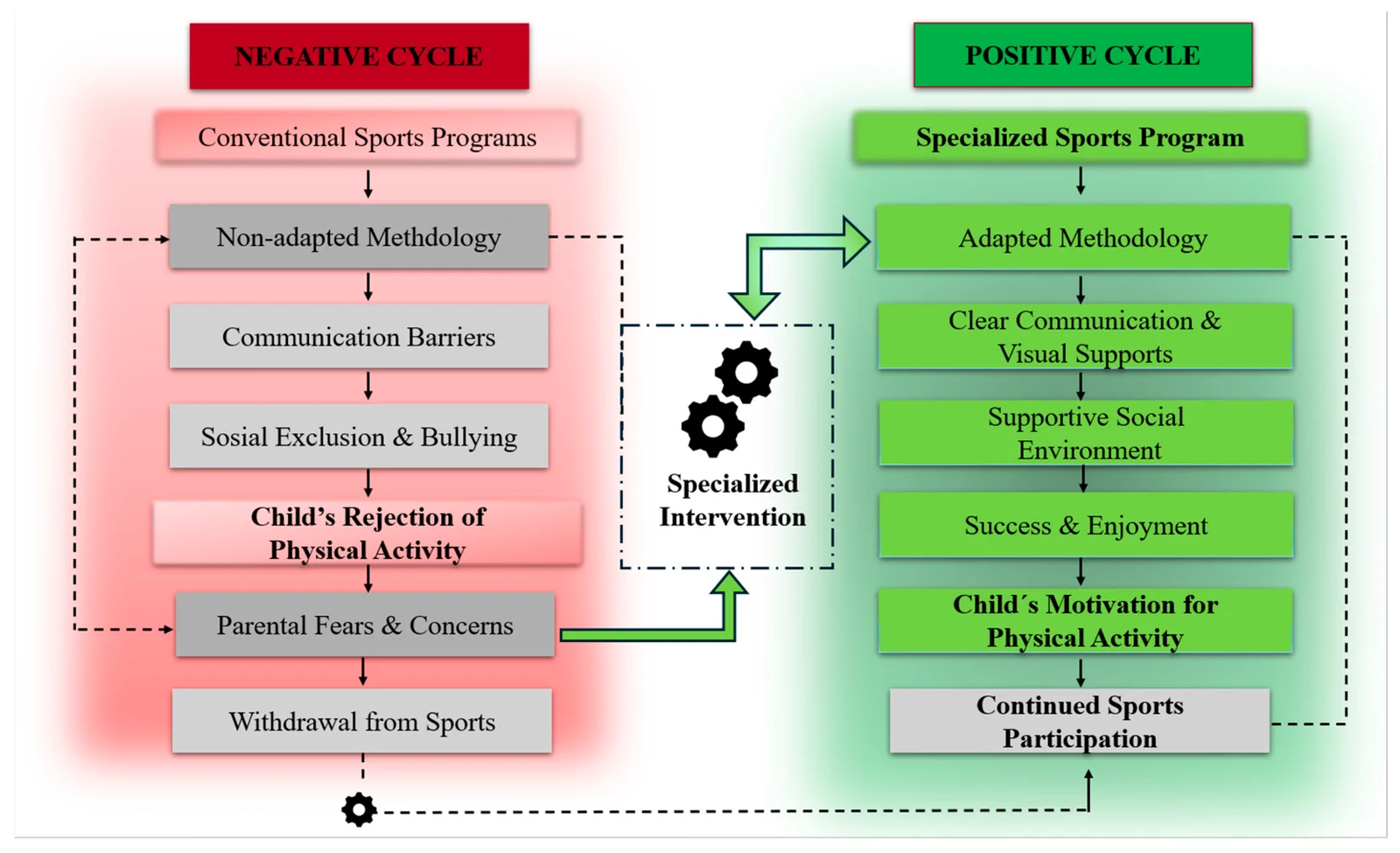
Parent-derived conceptual model of the trajectory from negative to positive physical activity experiences in boys with ASD Level 1.

Figure 2 presents a conceptual model synthesizing the trajectory described by parents, illustrating how boys with ASD Level 1 moved, in their parents’ accounts, from a negative cycle, characterized by exclusion, rejection, and accumulated failure in conventional sports settings, to a positive cycle of engagement, motivation, and continued participation following their involvement in the MCPAP. The model integrates the seven themes described above and identifies adapted instruction, trained professionals, and supportive environments as central elements in the transition from disengagement to engagement. The figure represents a parent-derived conceptual interpretation, not a demonstrated causal pathway.

## 4. Discussion

This qualitative study explored parental perspectives on barriers and facilitators to sports participation in boys with ASD Level 1 following their involvement in a multicomponent physical activity program. Findings illuminate the complex interplay of individual, social, and systemic factors that shape physical activity engagement for this population and identify both the obstacles that hinder participation in conventional sports settings and the program elements that supported engagement and positive change in the MCPAP.

### 4.1. Boys’ Rejection as a Parent-Perceived Response to Social Exclusion

The most notable finding of this study is the distinction parents drew between their sons’ apparent disinterest in physical activity and what they perceived as aversion resulting from negative social and sporting experiences. This parent-derived interpretation challenges assumptions, sometimes held by parents themselves prior to program participation, that boys with ASD are fundamentally disinclined toward physical activity. Previous research has documented lower physical activity levels among children with ASD [5,6], but the underlying mechanisms have remained unclear. Parents in our study consistently interpreted environmental and social factors, particularly bullying, social exclusion, and experiences of failure, as central to their sons’ attitudes toward sports, although this does not exclude possible interactions with autism-related characteristics, motor difficulties, sensory factors, or communication demands.

This interpretation aligns with research highlighting the significant impact of bullying on children with ASD, who experience victimization at elevated rates compared to typically developing peers [15,16]. The barriers identified in our study align with those documented by Obrusnikova and Cavalier [11], who found that perceived barriers to after-school physical activity in children with ASD include social challenges and environmental factors. Based on these findings, sports settings may be particularly high-risk due to their competitive nature, emphasis on social interaction, and exposure of individual differences in motor ability. When children experience mockery, rejection, or failure in sports settings, parents perceived that their children may develop avoidance responses that generalize across physical activity contexts.

Importantly, parents perceived that, following participation in the MCPAP, their sons were supported in overcoming and, in some cases, reducing their previous avoidance of physical activity through positive experiences in adapted environments. Many boys expressed enthusiasm for continued sports involvement and successfully transitioned to community activities such as karate, athletics, and soccer. This observation aligns with evidence that exercise-based interventions can produce meaningful behavioral changes in children and youth with ASD [31] and is consistent with parents’ accounts suggesting that interventions addressing the social and instructional environment may help reengage boys who have previously withdrawn from physical activity.

### 4.2. The “Invisible Population” of ASD Level 1

Parents in this study described their sons with ASD Level 1 as an “invisible population,” inadequately served by existing service structures that typically distinguish between mainstream programs and those designed for individuals with significant disabilities. This finding resonates with broader literature on the challenges faced by individuals with less visible support needs, who may be overlooked or misunderstood due to the absence of obvious impairment [22,23].

Children with ASD Level 1 often have sufficient cognitive and language abilities to participate in mainstream settings, but their social communication difficulties and sensory sensitivities generate support needs that are often unmet [22]. These challenges include impairments in pragmatic and contextual language use and conversation, including difficulties with topic maintenance, perseveration, and providing relevant contributions during dialogue [20], which can affect the quality of peer interaction even when structural language abilities are intact [21].

In sports contexts, these children may be expected to function without accommodation while simultaneously encountering environments ill-suited to their needs. The result, as parents in our study described, is a “limbo” in which children fit neither mainstream nor specialized programs. This finding is consistent with Gregor et al. [17], who documented Canadian parents’ perspectives on the significant challenges their adolescents with ASD face in accessing physical activity opportunities. Similarly, Obrusnikova and Miccinello [24] identified multiple parental concerns regarding factors influencing after-school physical activity participation.

This service gap has implications for policy and practice. There is a need for programs specifically designed for children with ASD Level 1 that recognize their unique profile of abilities and support needs. Additionally, efforts to build capacity within mainstream sports organizations to accommodate children with milder forms of ASD could expand participation opportunities. We note that the characterization of children with ASD Level 1 as an “invisible population” is a parent-derived concept and should not be generalized to all children with ASD Level 1 without further evidence.

### 4.3. Importance of Specialized Professionals

Parents consistently emphasized the critical role of professionals trained to understand and support children with ASD. This finding corroborates previous research identifying coach training as a key factor in successful sports participation for children with disabilities [14,25,30]. Specific competencies valued by parents included: understanding behavioral presentations as expressions of underlying difficulties, employing adapted communication strategies, implementing effective redirection techniques, and creating predictable and supportive environments.

The contrast between parents’ experiences with untrained professionals in conventional settings and the MCPAP instructor was striking. Previous negative experiences had often involved professionals who lacked understanding of ASD and responded to behavioral challenges in ways that exacerbated difficulties. In contrast, the MCPAP instructor’s expertise enabled proactive prevention of difficulties and constructive responses when challenges arose.

These findings highlight the need for professional development programs that equip sports instructors with knowledge and skills for supporting children with ASD. Such training should address both theoretical understanding of autism and practical strategies for inclusive instruction.

### 4.4. Parent-Reported Outcomes and Transfer to Natural Contexts

The seventh theme of this study identified a range of parent-reported positive outcomes associated with the MCPAP, including perceived improvements in behavioral and emotional regulation, the parent-reported transfer of gains to school and natural contexts, a reduction in avoidance, and broader well-being and family impact. These accounts are consistent with prior intervention research documenting the regulatory and behavioral effects of structured physical activity in children with ASD [2,3,4,31], and suggest that such effects, when they occur, may be observable by parents in everyday family life and not only in standardized outcome measures. A particularly important form of transfer involved parental mediation of program strategies into the school context, with one mother explaining how she shared the MCPAP approach with her child’s physical education teacher, which she reported as leading to improvements in school participation. The formation of an unintended peer-support network among mothers is also noteworthy and suggests that programs of this kind may yield benefits for family functioning that extend beyond the participating child. It is important to note that these outcomes reflect parental perception and report rather than standardized measurement; parents are nonetheless uniquely positioned to detect subtle changes in affect, motivation, and behavior across settings, although their accounts are also subject to retrospective attribution and social desirability biases.

### 4.5. Methodological Considerations

Parents’ observations regarding methodological barriers align with evidence-based recommendations for autism-inclusive physical activity instruction [14,30,31]. Key principles include simplified and explicit verbal instructions, visual support, structured and predictable formats, small group sizes, and a focus on enjoyment rather than competition. MCPAP embodied these principles, and parents attributed much of its success to these methodological features.

Conventional sports programs often rely on implicit social learning, complex verbal instructions, and large group formats that disadvantage children with ASD. Adapting instructional methodologies need not require separate programs; rather, inclusive practices can be implemented within mainstream settings when professionals have appropriate training and support. These methodological principles are supported by intervention research documenting the effects of structured physical activity programs on children with ASD [2,3,4].

An additional design feature of the MCPAP warranting attention was the deliberate inclusion of two to three neurotypically developing peers per group. This composition reflects a peer-mediated approach that has shown promise in supporting social engagement among children with ASD [30]. Parents did not comment on this feature extensively, which may reflect its integration as a normalized rather than distinctive element of the program; future research should explicitly examine how typically developing peers experience and contribute to inclusive sports groups.

### 4.6. Parental Fears as Barriers

The significant role of parental apprehension in shaping children’s access to sports activities warrants attention. Obrusnikova and Miccinello [24] similarly documented that parental perceptions significantly influence children’s access to physical activity opportunities. Parents described fears related to failure, rejection, bullying, and lack of understanding that often preceded and exceeded any difficulties their children actually encountered. These anticipatory concerns functioned as psychological barriers that could limit children’s opportunities regardless of program quality.

Salar et al. [36] identified family-related factors, such as parental apprehension and limited scheduling capacity, as primary barriers to PA, underscoring how domestic perceptions directly constrain children’s opportunities. While such protective impulses are understandable given children’s vulnerabilities, they may inadvertently limit opportunities for positive experiences and skill development. Interventions that address parental concerns and provide reassurance through program design features may help overcome these barriers.

### 4.7. Implications for Practice

The findings of this study have several implications for sports organizations, healthcare professionals, and policymakers. First, sports programs serving boys with ASD Level 1 should be designed and delivered by professionals with specific training in autism, including the ability to recognize behavioral presentations as expressions of underlying needs, to implement adapted instructional strategies, and to provide predictable, supportive environments. Second, the small-group, structured format that characterized the MCPAP, rather than a fixed curriculum or specific sport, appears to be a key facilitator of engagement, suggesting that this design principle could be transferred to mainstream sports organizations. Third, given the centrality of parental fears as a barrier, sports organizations could implement pre-program orientations and ongoing communication with families to address anticipatory concerns and build trust. Fourth, the geographic concentration of specialized programs in urban centers represents an equity issue that policymakers could address through funding for decentralized service delivery, including school-based and community-club partnerships. Finally, the positive outcomes reported by families in natural contexts suggest that programs should be evaluated not only on immediate engagement but also on broader indicators of transfer, well-being, and family impact.

### 4.8. Limitations and Future Directions

Several limitations should be considered when interpreting these findings. First, the study focused exclusively on parental perspectives; the voices of children with ASD and their siblings were not directly represented. Future research should incorporate child perspectives to provide a more complete understanding of barriers and facilitators. Second, participants were parents of boys who had completed the MCPAP, potentially introducing selection bias toward families with positive program experiences. Parents who withdrew their children or chose not to participate may have different perspectives. Third, the study sample was restricted to parents of boys with ASD Level 1, as the MCPAP cohort comprised exclusively male participants during the study period. This represents an important limitation, as girls with ASD may experience distinct patterns of social and motor challenges, including different peer interaction dynamics in sports settings. Fourth, all outcomes reported in this study are parent-reported and were not triangulated with standardized measures or teacher reports (with the exception of a single teacher-mediated observation reported by one parent); future research should incorporate multiple measurement modalities. Fifth, interviews were conducted up to approximately 19 months after program completion for some families, and the parent-reported outcomes should be interpreted with caution in light of potential retrospective attribution bias, social desirability bias, and the influence of researchers’ positionality. The first author’s dual role as program facilitator and analyst may have shaped the interpretation of program-related findings, although the use of an external interviewer, reflexive journaling, independent coding by a second researcher, and peer debriefing were implemented to mitigate this risk. Finally, the study was conducted in a single geographic and cultural context (Valencia, Spain), limiting generalizability to other settings.

Future research should explore the perspectives of diverse stakeholders, including children themselves and their siblings; examine barriers and facilitators across different cultural contexts; investigate the long-term impact of positive sports experiences on children’s physical activity trajectories; and purposively recruit families of girls with ASD Level 1, who were not represented in the present sample.

## 5. Conclusions

This study contributes to the limited literature on the specific experiences of boys with ASD Level 1 in sports contexts by foregrounding parental perspectives on barriers, facilitators, and outcomes. The parent-derived interpretation that boys’ apparent rejection of physical activity reflects responses to negative social experiences rather than inherent disinterest offers an alternative to deficit-oriented assumptions that have historically shaped research and program design for this population. Parents described children with ASD Level 1 as occupying a service gap that current programs fail to address, suggesting that the development of specialized sports programs, with trained professionals, evidence-based instructional adaptations, and family-centered approaches, is needed to support this underserved population. The parent-reported outcomes observed in this study suggest that interventions addressing the social and instructional environment, rather than attempting to modify characteristics of the children themselves, may promote sustained engagement in physical activity and behavioral changes that extend into natural contexts. These interpretations, however, reflect parental perceptions and were not triangulated with standardized outcome measures; further research is needed to test these parent-derived hypotheses through mixed methods and longitudinal designs.

## Figures and Tables

**Table 1:** Participant characteristics.

Code	Parent	Parent Age	Education Level	Employment Status	Child Age
E1	Father	46	Bachelor’s degree	Full-time employed	12
E2	Father	48	Secondary	Full-time employed	9
E3	Father	45	Bachelor’s degree	Full-time employed	9
E4	Father	49	Bachelor’s degree	Full-time employed	9
E8	Father	46	Bachelor’s degree	Full-time employed	10
E9	Father	51	Bachelor’s degree	Full-time employed	10
E12	Mother	49	Bachelor’s degree	Full-time employed	12
E13	Mother	47	Bachelor’s degree	Full-time employed	9
E14	Mother	42	Bachelor’s degree	Homemaker/Unemployed	9
E15	Mother	49	Bachelor’s degree	Full-time employed	9
E16	Mother	47	Bachelor’s degree	Full-time employed	12
E17	Mother	43	Secondary	Full-time employed	10
E18	Mother	42	Secondary	Homemaker/Unemployed	12
E19	Mother	41	Bachelor’s degree	Full-time employed	10
E20	Mother	51	Bachelor’s degree	Full-time employed	10
E21	Mother	45	Bachelor’s degree	Full-time employed	9
E22	Mother	44	Bachelor’s degree	Full-time employed	10

**Table 2:** Themes and subthemes identified in the analysis.

Theme	Subtheme	Description
1. Subjective Barriers Rooted in Parental Fears	Fear of failure and rejection	Concerns that children would be unable to meet demands and experience frustration
	Fear of social difficulties	Worries about bullying, mockery, and social exclusion
	Fear of incomprehension	Concerns that coaches would not understand the child’s needs or behaviours
2. Children’s Rejection as Response to Social Exclusion	Bullying and peer rejection	Experiences of mockery and exclusion in sports settings
	Accumulated negative experiences	Multiple failed attempts creating lasting aversions
	Rejection of context, not activity	Distinction between rejecting social circumstances versus physical activity itself
3. Methodological Barriers in Conventional Settings	Adapted communication and structured delivery	Verbal complexity, implicit expectations and lack of routine creating anxiety
	Group size and attention	Large groups limiting individualized support
4. Challenges with Non-Specialized Professionals	Understanding behavioral presentations	Recognizing behaviours as expressions of underlying difficulties
	Adapted communication and redirection	Effective strategies for managing challenges
	Expertise as essential	ASD knowledge distinguished successful experiences
5. Scarcity of Specific Programs for ASD Level 1	The “invisible population”	Children falling between mainstream and specialized services
	Lack of systemic guidance	Families navigating services without adequate support
	Need for continued initiatives	Desire for program expansion and continuity
6. Location and Accessibility Concerns	Proximity to home or school	Transportation and access challenges
7. Facilitators and Positive Outcomes Associated with the MCPAP	Behavioural and emotional regulation	Improvements in self-regulation, calmness, and emotional expression during sessions
	Transfer to school and natural contexts	Generalization of gains to school playground, classroom, and peer interactions
	Reduced avoidance and continued engagement	Transition to other sports and continued physical activity post-program
	Broader well-being and family impact	Improvements in sleep, family communication, and parental peer support

## Data Availability

The Codebook and interview guide used in this study are publicly available at https://osf.io/zdvhb/overview?view_only=047d3610c1194b938e7491081a988dc1 (accessed on 8 August 2026). The interview transcripts are not publicly available to protect participant confidentiality but may be made available by the corresponding author upon reasonable request, subject to ethical approval and applicable data protection requirements.

## Abbreviations

The following abbreviations are used in this manuscript:

ASD	Autism Spectrum Disorder
ASD Level 1	Autism Spectrum Disorder Level 1 (requiring support)
MCPAP	Multicomponent Physical Activity Program
ADI-R	Autism Diagnostic Interview-Revised
ADOS	Autism Diagnostic Observation Schedule
DSM-5	Diagnostic and Statistical Manual of Mental Disorders, fifth edition
TEACCH	Treatment and Education of Autistic and Related Communication-Handicapped Children
COREQ	Consolidated Criteria for Reporting Qualitative Research
